# First-Principles Study on the Stability and STM Image of Borophene

**DOI:** 10.1186/s11671-017-2282-7

**Published:** 2017-08-29

**Authors:** Zhifen Luo, Xiaoli Fan, Yurong An

**Affiliations:** State Key Laboratory of Solidification Processing, School of Material Science and Engineering, Northwestern Polytechnical University, 127 YouYi Western Road, Xi’an, Shaanxi 710072 China

**Keywords:** Atomic-thin boron sheet, First-principles calculations, Atomic structure, Stability, STM image

## Abstract

Very recently, borophene (atomic-thin two-dimensional boron sheet) has been successfully synthesized on the Ag(111) surface by deposition. Two kinds of structures were found. However, the identification of the monolayer boron sheets grown on the metal substrate, as well as the stability of different 2D boron sheets, is controversial. By performing the first-principles calculations, present study investigates the atomic structure, stability, and electronic properties of the most possible boron sheets grown on metal surface, namely, buckled triangular, β_12_, and χ_3_ types of crystal lattice. Our result shows that all the three freestanding sheets are thermodynamically unstable and all are metallic. On the other hand, our result indicates the Ag(111) substrate stabilize these sheets. Additionally, our simulated STM images of these monoatomic-thin boron sheets on Ag(111) surface reproduce the experiment observations well and clearly identify the as-grown boron sheets.

## Background

Since the discovery of graphene, two-dimensional (2D) materials have become one of the most active nanomaterials due to their unique physical properties and potential applications in next-generation electronics and energy conversion device [1–7]. Recently, a class of 2D boron nanostructures has been discovered and attracted significant attentions [8–21]. However, there was no evidence that the 2D boron sheets could be realized experimentally until very recently, both Mannix et al. [22] and Feng et al. [23] made spectacular advances in realizing experimentally the atomic-thin 2D boron sheets. The extended 2D boron sheet is called “borophene”, in analogy to graphene.

During the last two decades, numerous 2D boron nanostructures have been discovered [8–21]. Besides the hexagonal sheet and the triangular sheet [20, 21], as well as the buckled triangular sheets [8], other 2D boron sheets with hexagonal holes, like the *α*-sheet [9, 18], *β*-sheet [9, 18], *γ*-sheet [19], and g1/8 and g2/15 sheets [15], were examined by the ab initio calculations. It was suggested that the triangular planar boron lattice with hexagonal vacancies is more stable [9]. And a variety of such triangular boron layer with different patterns of hexagonal holes were reported by both the computational and experimental research groups [11, 13–16]. However, all of these monoatomic-thin boron layers are higher in energy than boron’s three-dimensional (3D) bulk state, which means that the 2D structure of boron is disadvantaged thermodynamically. Thus, a sufficiently “sticky” substrate is necessary to suppress the 3D nucleation barrier to entice the atoms into the 2D route.

Recently, the formation of boron sheets on metal and metal boride substrates has been explored by first-principles calculations [24]; it suggests that the boron sheets can be grown on the Ag(111) and Au(111) surface. Additionally, Piazza et al.’s [14] study provide experimental evidence that the monolayer boron sheets are achievable based on their observations of B_36_ cluster; it was shown to be a highly stable planar cluster with a central hexagonal hole [14]. More recently, two groups [22, 23] successfully synthesized the atomic-thin, crystalline 2D boron sheets on a silver surface by directly evaporating a pure boron source via molecular beam epitaxy.

Mannix et al. [22] found two distinct phases of the boron sheet on silver substrate using high-resolution scanning tunneling microscopy (STM) characterization: a striped phase and a homogeneous phase. Feng et al. [23] also found two phases of boron sheet, which look quite similar to those reported in Mannix et al.’s report, and they described the homogeneous phase with zigzag rows of protrusions as χ_3_ lattice of boron sheet. On the other hand, their interpretations for the stripe phase are quite different. Mannix et al. [22] assigned the striped phase as a buckled triangular lattice without vacancy. But Feng et al. [23] proposed the stripe phase to be the rectangular lattice displaying parallel rows of hexagonal holes, which was known as the β_12_ sheet.

The exact configurations and properties, as well as the applications of these 2D boron sheets, have attracted tremendous attentions [19, 22, 24, 25]. It was reported that the buckled triangular borophene is a highly anisotropic metal with a high Young modulus along its armchair direction which exceeds that of graphene [22]. Sun et al. also found that the lattice thermal conductivity of the buckled triangular borophene is strongly anisotropic [26]. Moreover, Gao et al. reported that the β_12_ borophene and χ_3_ borophene may be another superconducting phase of boron besides MgB_2_ thin film [27]. However, the thermodynamic stability of β_12_ borophene and χ_3_ borophene are controversial [27, 28]. According to Gao et al.’s study, both β_12_ borophene and χ_3_ borophene are stable [27]. But Penev et al. reported that both β_12_ borophene and χ_3_ borophene have imaginary frequencies near the G point in their phonon spectrums [28].

To provide a better understanding for the experimental achievable borophene, we systematically investigated the possible atomic structures and their stability, as well as the electronic properties by performing the first-principles calculations. Our results indicate that β_12_ and χ_3_ sheets are thermodynamically unstable. Additionally, the configurations of buckled triangular, β_12_, and χ_3_ sheets all show metallic feature. Moreover, we have simulated the STM images for the freestanding and epitaxial monolayer of boron on the Ag(111) surface; we found buckled triangular and β_12_ boron sheets on Ag(111) surface both look as stripe phases but with little difference.

## Computational Methods

The calculations are performed by using the Vienna ab-initio simulation package (VASP) based on density functional theory (DFT) [29, 30]. The projector-augmented-wave method was adopted for the calculations of electron–ion interactions [31, 32]. And the electronic exchange–correlation interactions were described by the generalized gradient approximation (GGA) using the Perdew–Burke–Ernzerhof (PBE) functional [33]. Wave functions were expanded in a plane wave basis with an energy cutoff of 500 eV. The first Brillion zone were sampled by 25 × 15 × 1, 15 × 9 × 1, and 11 × 11 × 1 k-mesh for the buckled triangular, β_12_, and χ_3_ phases of borophene, respectively. To simulate the 2D boron sheets, a vacuum space of at least 20 Å is included along the Z direction to minimize the interaction between the periodic images. The convergence criterion was set to 10^−5^ eV between two ionic steps for the self-consistency process. All structures were fully relaxed until the force on each atom was less than 0.02 eV Å^−1^, and the bottom two layers of silver atoms were fixed. Phonon dispersion spectrums have been computed by using the finite displacement method as implemented in the PHONOPY package [34].

The STM images were simulated using the Tersoff–Hamann formula and its extension [35]. Briefly, assuming that the density of states of the tip is constant, we can approximate the STM tunneling current with the local density of states, $\rho \left(\overrightarrow{r},E\right)$, as the only variable with the following expression:

$$ I(V)\propto {\int}_{E_{\mathrm{F}}}^{E_{\mathrm{F}}+ eV}\rho \left(\overrightarrow{r},E\right) dE $$

$$ \rho \left(\overrightarrow{r},E\right)=\sum_i\left|{\psi}_i{\left(\overrightarrow{r}\right)}^2\right|\delta \left(E-{E}_i\right) $$

where $\rho \left(\overrightarrow{r},E\right)$ is the LDOS on the sample surface, ${\psi}_i\left(\overrightarrow{r}\right)$is the sample wave function with energy *E*_*i*_, and *E*_F_ is the Fermi energy. When the states in $\rho \left(\overrightarrow{r},E\right)$ are filled, it is also common to refer to $\rho \left(\overrightarrow{r},E\right)$ as the charge density of the states. The simulated STM images were obtained using the constant current mode based on calculated electron densities.

## Results and Discussion

**Fig. 1:**
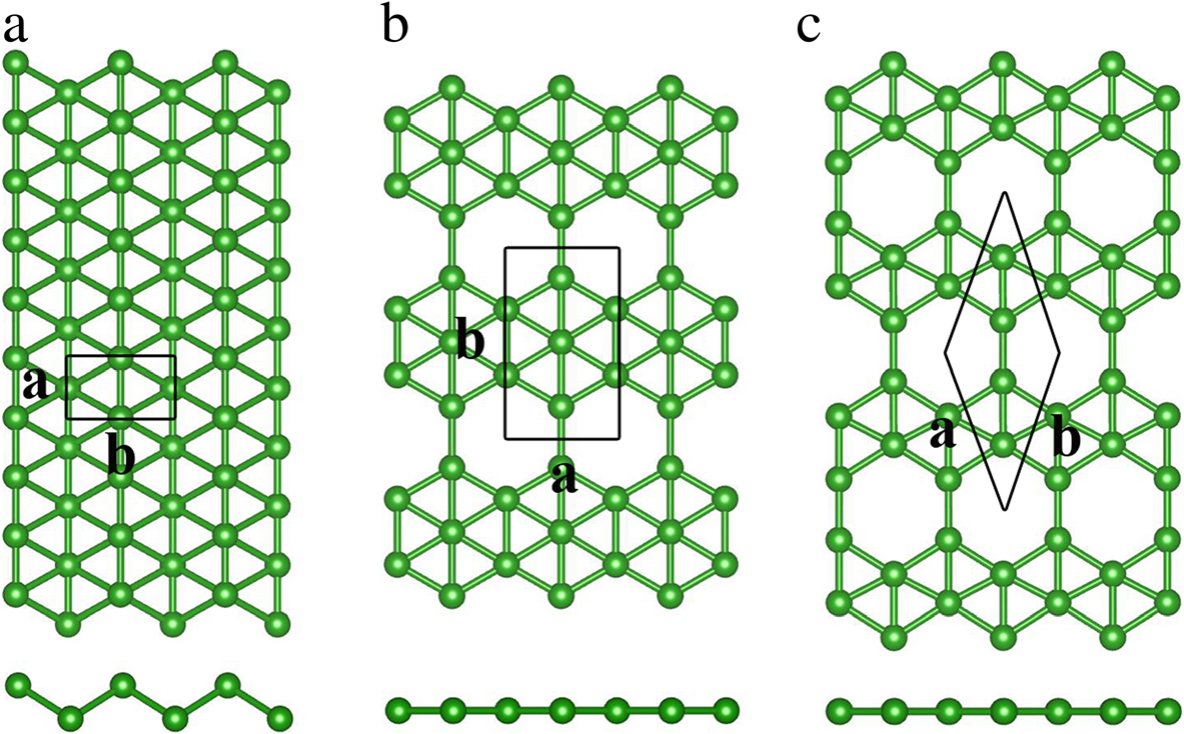
Top and side views of buckled triangular (**a**), β_12_ (**b**), and χ_3_ (**c**) boron sheets. The green balls represent the boron atoms. The rectangles and rhombus enclosed by solid black lines denote the unit cells. The letters *a* and *b* represent the lattice parameter

Figure 1 shows our calculated results for the buckled triangular, β_12_, and χ_3_ lattice structures of borophene. Unlike the one-atom thin and planar hexagonal configuration of graphene, buckled triangular borophene shows buckling along one lattice direction. On the other hand, the structures of β_12_ and χ_3_ borophenes are planar without out-of-plane buckling. Figure 1a shows that there are two boron atoms in the unit cell of buckled triangular borophene. And the space group of buckled triangular borophene is Pmmn. Our optimized lattice constants are *a* = 1.613 Å and *b* = 2.866 Å, agreeing well with previous theoretical and experimental results [22]. The β_12_ borophene shown in Fig. 1b has filled and empty hexagons along the zigzag direction; the corresponding space group is P2mm. There are five boron atoms in the unit cell. The lattice constants are 2.916 and 5.075 Å along the *a* and *b* directions. The unit cell of χ_3_ borophene is rhombic, having four boron atoms and the lattice constant of 4.448 Å. Its space group is C2mm. Table 1 lists our calculation results on the lattice constants, which agree well with previous results [22, 23, 27, 36].

**Table 1:** Our calculated lattice constants *a*, *b*, and *γ* for the buckled triangular, *β*_12_ and *χ*_3_ boron sheets. Previous results are also listed for comparison

	Previous studies			Present result		
	*a* (Å)	*b* (Å)	*γ*	*a* (Å)	*b* (Å)	*γ*
b_△_	1.617 [22]	2.872 [22]	90°	1.613	2.866	90°
β_12_	2.922 [23]	5.057 [23]	90°	2.916	5.075	90°
χ_3_	4.443 [27]	4.443 [27]	38.173° [27]	4.448	4.448	38.182°

As shown in Fig. 1, there are vacancies in both β_12_ and χ_3_ sheets but not in the buckled triangular lattice and the number of vacancies in β_12_ and χ_3_ borophene is different. The vacancy concentration *η* is defined as the ratio between the number of vacancy sites and total sites (including vacancy) in the unit cell; it is a quantity describing the boron sheets from global and local points of view [9]. *η* is 1/6 in β_12_ lattice and 1/5 in χ_3_ lattice. Compared to the β_12_ lattice, Fig. 1c shows that the neighboring vacancy rows in χ_3_ borophene are shifted by half of the lattice constant in zigzag direction, resulting in a planar of C2mm symmetry.

We calculate the average energy of each boron atom using the following equation for the three structures and use it to compare the relative stability of the three structures; this method has been applied in Ref. [23]

$$ {E}_{\mathrm{FB}}={E}_{\mathrm{borophene}}/n $$

where *E*_borophene_ and *n* are the energy and the number of boron atoms in one unit cell, respectively. Our calculated results are summarized in Table 2. It indicates that the β_12_ phase is the most stable, while the χ_3_ phase is the least stable with relative higher energy of 0.08 eV.

**Table 2:** Average energy for boron atoms in the freestanding (*E*_FB_) and epitaxial (*E*_EB_) boron sheets on the Ag(111)

	Previous studies			Present result			
	b_△_	β_12_	χ_3_	b_△_	β_12_	χ_3_	χ_3_’
E_FB_(eV/atom)	/	− 6.23 [23]	− 6.19 [23]	− 6.19	− 6.23	− 6.15	− 6.15
E_EB_(eV/atom)	/	− 6.32 [23]	− 6.35 [23]	− 6.29	− 6.33	− 6.32	− 6.35

The results for the buckled triangular, β_12_, and χ_3_ boron sheets are listed

**Fig. 2:**
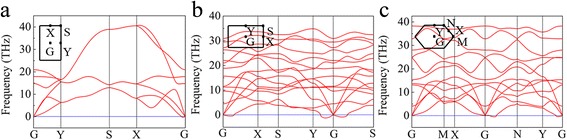
The phonon dispersion of the **a** buckled triangular, **b** β_12_, and **c** χ_3_ boron sheets. The high symmetry points are shown on the left corner

We then calculated the phonon dispersion spectrum for the three phases of buckled triangular, β_12_, and χ_3_ borophene. Figure 2 shows the phonon dispersion spectrums along the high symmetry directions. As shown in Fig. 2a, there are three acoustic and three optical phonon branches for the buckled triangular borophene. It also shows imaginary values near the G point along the X–G direction, indicating that the lattice is unstable along the *a* direction, which explains the stripe formed along the *a* direction in the experimental STM images [23]. In fact, recent studies have suggested that the biaxial tensile and uniaxial tensile cannot stabilize the freestanding buckled triangular borophene even under the tensile stress of 0.08% [36, 37]. Figure 2b, c shows that there are also imaginary frequencies near the G point of β_12_ and χ_3_ phases. Our results show that all the three phases of buckled triangular, β_12_, and χ_3_ are unstable.

**Fig. 3:**
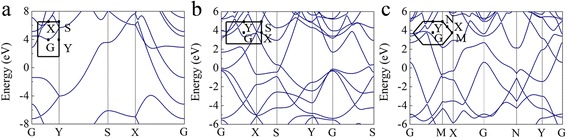
Calculated band structures for **a** buckled triangular, **b** β_12_, and **c** χ_3_ boron sheets. The Fermi energy was set to zero. The high-symmetry points are shown on the left corner

We further studied the electronic structures of buckled triangular borophene, β_12_ borophene, and χ_3_ borophene. The calculated band structures along the high-symmetry directions are shown in Fig. 3. As shown in Fig. 3, all the three phases of buckled triangular, β_12_, and χ_3_ borophenes are metallic. Particularly, for the buckled triangular borophene as shown in Fig. 3a, three energy bands cross the Fermi level: one is along the S–Y direction and the other two are along the G–X direction. However, we have mentioned in the above sections that the buckled triangular is buckling along the *b* direction, which opens a bandgap of 9.63 and 4.32 eV along the X–S and Y–G directions, respectively. It indicates that the buckled triangular borophene behaves as a metal with strong anisotropy and the electrical conductivity is confined along the uncorrugated *a* direction.

**Fig. 4:**
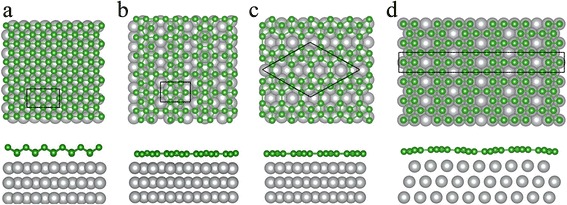
Top and side views of boron sheets on Ag(111) surface. **a** Buckled triangular, **b** β_12_, **c** χ_3_, and **d** χ_3_’ boron sheet. The green and gray balls represent boron and silver atoms, respectively. The rectangles and rhombus enclosed by solid black lines denote the unit cells of boron sheets on the Ag(111) surface

Additionally, we studied the atomic structures and stability of the buckled triangular, β_12_, and χ_3_ boron sheets on the Ag(111) substrate. The results are shown in Fig. 4. The unit cell of buckled triangular borophene on Ag(111) surface is the (1 × 3) supercell of freestanding buckled triangular borophene and the rectangular 1 × (√3)R30° supercell of the Ag(111) substrate. For the configuration of β_12_ sheet on the Ag(111) surface, the unit cell is the unit cell of β_12_ borophene and 1 × (√3)R30° supercell of the Ag(111) surface. Our calculations show that the β_12_ borophene matches to Ag(111) surface (~ 1% mismatch) better than the buckled triangular borophene (~ 3% mismatch). The χ_3_ borophene forms two configurations on the Ag(111) surface, as shown in Fig. 4c, d, which are named as χ_3_ and χ_3_’. The unit cell of χ_3_ is rhombus with lattice constant of *a* = 8.67 Å, and the unit cell of χ_3_’ is orthorhombic with lattice parameters of *a* = 2.89 Å and *b* = 25.02 Å; it is the 1 × (5√3)R30° supercell of the Ag(111) surface.

According to our calculations, the vertical distances from the Ag(111) surface to the lower and upper boron atomic layers of the buckled triangular borophene are 2.5 and 3.3 Å, respectively, indicating the weak interaction between the boron sheet and the Ag substrate. The β_12_, χ_3_, and χ_3_’ sheets all remain planar on the Ag(111) surface, and the vertical distances between the boron sheet and the Ag surface are 2.4~2.9 Å. The results agree with the measured thickness of ~ 2.7 to 3.1 Å reported by Mannix et al. [22]. We compared the atomic structures of buckled triangular, β_12_, χ_3_, and χ_3_’ phases of borophene on Ag substrate with the counterparts of the freestanding borophene and found that these four structures change little. The buckling height *h* of buckled triangular borophene is shorter from 0.910 to 0.857 Å, and the B-B lengths are longer about 0.1 Å. Moreover, the hexagon vacancies in the β_12_ borophene shrink along a direction, and those in χ_3_ borophene become little larger.

Similar to the calculation for the relative stability of the freestanding borophene, we further calculated the average energy of each boron atom for the boron sheets on the Ag(111) surface via the following formula:

$$ {E}_{\mathrm{EB}}=\frac{1}{n}\left({E}_{\mathrm{tot}}-{E}_{\mathrm{sub}}\right) $$

where *E*_tot_ is the total energy of the boron sheet and the Ag(111) surface, *E*_sub_ is the energy of Ag substrate, and *n*is the number of boron atoms in one unit cell. Our result shows that the possibility of forming buckled triangular, β_12_, χ_3_, and χ_3_’ lattices on the Ag(111) surface is similar based on their close energies. Additionally, the energies of the borophene on the Ag(111) surface are lower by 0.1~0.2 eV per boron atom relative to the freestanding sheets. This means that Ag(111) surface stabilize the borophene.

**Fig. 5:**
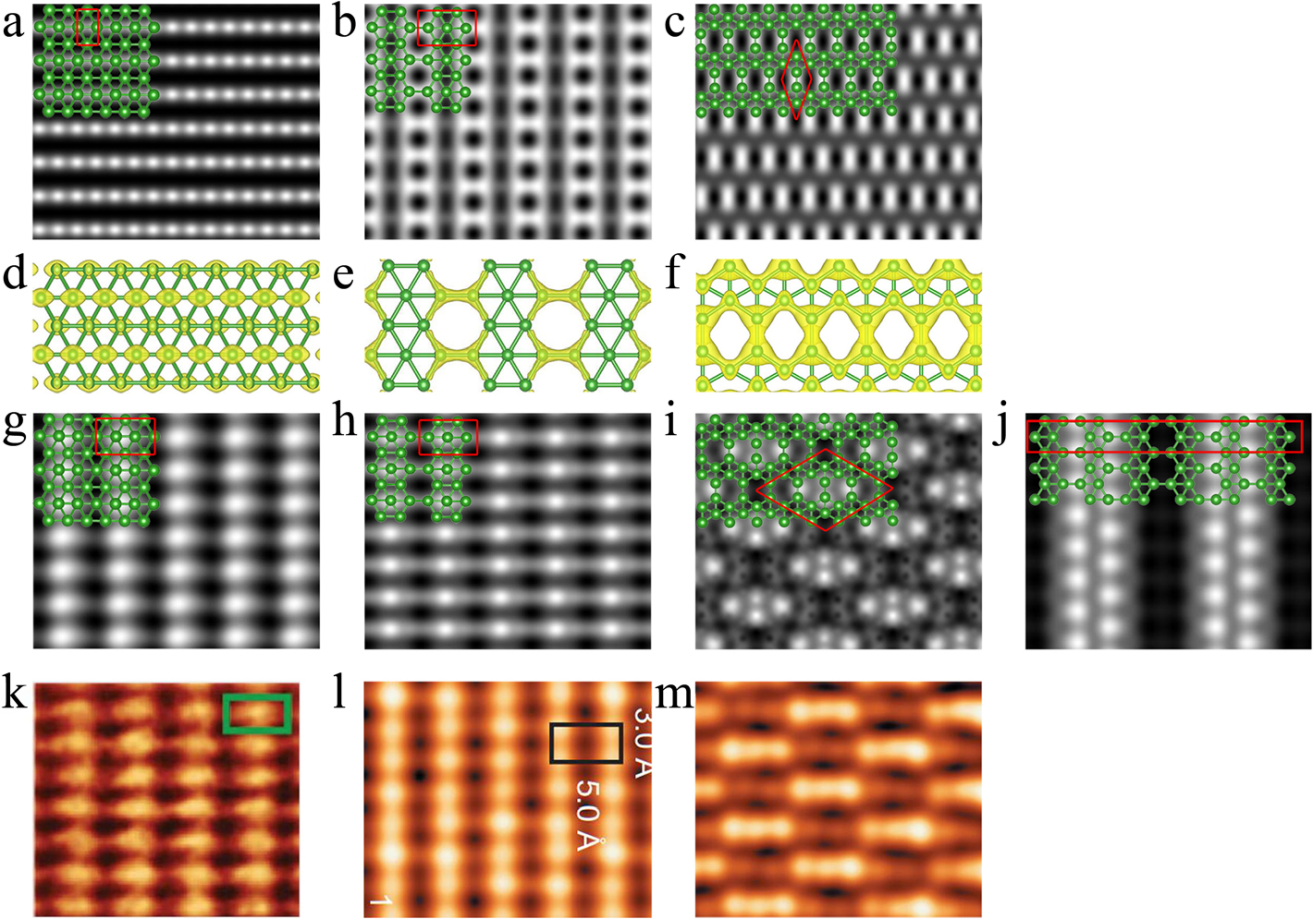
Simulated STM images of freestanding and epitaxial boron sheets on Ag(111) surface. Freestanding **a** triangular, **b** β_12_, and **c** χ_3_ boron sheets. Partial charge density of freestanding **d** triangular, **e** β_12_, and **f** χ_3_ boron sheets. **g** Buckled triangular, **h** β_12_, **i** χ_3_, and **j** χ_3_’ boron sheet on the Ag(111) surface. The bias voltage is 1.0 V. The green balls represent the boron atoms. The rectangles and rhombus enclosed by solid red lines denote the unit cells of freestanding and as-grown boron sheets on Ag(111) surface, respectively. Experimentally observed **k** stripe phase in Ref. [22], **l** stripe phase in Ref. [23], and **m** homogeneous phase in Ref. [23]

Figure 5 shows our simulated STM images for the freestanding and as-grown boron sheets on Ag(111) surface, as well as the partial charge density for the freestanding boron sheets. As shown in Fig. 5a, the freestanding buckled triangular boron sheet features stripes of bright spots. Figure 5d indicates that the bright spots come from the p_z_ orbit of upper boron atoms. Figure 5b shows rows of dark round spots surrounded by bright hexagons. Obviously, the hexagonal vacancies in β_12_ lattice shown in Fig. 1b result in the dark spots, while the bright hexagons correspond to the *σ* orbits of boron atoms around the hexagon holes as shown in Fig. 5e. As shown in Fig. 5c, the χ_3_ sheet displays rhombohedra pattern of bright spots in dumbbell shape. These bright dumbbell spots actually are the p_z_ orbits of the two boron atoms and the *σ* orbits formed between them.

The boron sheets on the Ag substrate all have larger unit patterns relative to the freestanding ones because of the mismatches between the unit cells of borophene and Ag(111) surface. Figure 5g shows our simulated STM image for buckled triangular boron sheet on Ag(111) surface. It displays stripes of bright spots in spindle shape, which agree very well with experimental observations [22]. Comparing with the image of freestanding buckled triangular boron sheet shown in Fig. 5a, the unit cell of STM image of buckled triangular boron sheet on the Ag(111) surface increases to three times. And the shape changes to spindle from round. The STM image of β_12_ sheet on Ag(111) surface shown in Fig. 5h shows rows of dark oval spots surrounded by four bright spots on its four corners. Different from the image of freestanding β_12_ sheet shown in Fig. 5b, the bright spots come from the p_z_ orbits of the boron atoms in the center of hexagons. As shown in Fig. 5i, the χ_3_ sheet has a rhombohedra STM pattern which is in good agreement with the experimental observed S2 phase [23]. The group of bright spots in the rhombohedra unit cell corresponds to the *σ* orbits and p_z_ orbits of the higher boron atoms in the unit cell, while the other boron atoms are invisible because they are lower.

Mannix et al. [22] and Feng et al. [23] both reported the stripe phase for 2D boron sheets on the Ag(111) surface based on their STM observations, and both the STM images feature parallel rows of protrusions. However, the shape of the bright spots in the two experimental observations is different; they are spindle in Mannix et al.’s report [22] and oval in Feng et al.’s [23]. Our simulated STM images of buckled triangular and β_12_ boron sheets match very well with the experimental observed stripe phases in Ref. [22] and Ref. [23], respectively, and the images shown in Fig. 5g, h clearly reproduce the difference between the experimental observations of Mannix et al. [22] and Feng et al. [23]. It also provides us a way to distinguish the two lattice of buckled triangular and β_12_. As for the STM image of χ_3_ sheet on the Ag(111) surface, as shown in Fig. 5i, it agrees with the experimental observation [23], but our result indicates that the bright spots come from the boron atoms on the edge of hexagonal vacancies instead of the filled triangular area as indicated in Ref. [23].

**Fig. 6:**
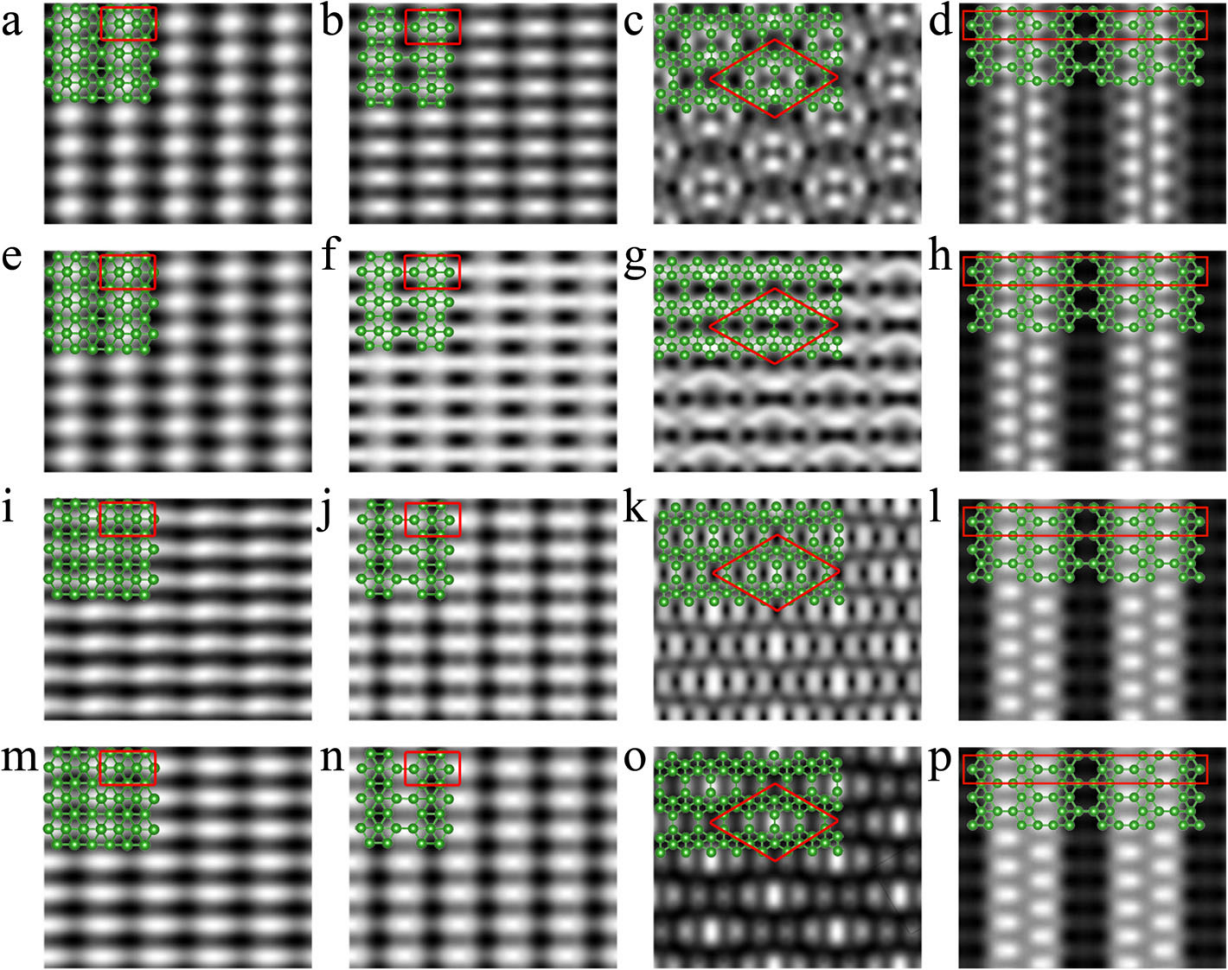
Simulated STM images for boron sheets on Ag(111). Buckled triangular borophene on Ag(111) at **a** 0.8, **e** 0.2, **i** − 0.4, and **m** − 1.0 V. β_12_ borophene on Ag(111) at **b** 0.8, **f** 0.2, **j** − 0.4, and **n** − 1.0 V. χ_3_ borophene on Ag(111) at **c** 0.8, **g** 0.2, **k** − 0.4, and **o** − 1.0 V. χ_3_’ borophene on Ag(111) at **d** 0.8, **h** 0.2, **l** − 0.4, and **p** − 1.0 V. The green balls represent the boron atoms. The rectangles and rhombus enclosed by solid red lines denote the unit cells of as-grown boron sheets on Ag(111) surface

In order to further distinguish the lattice structures of boron sheets on the Ag(111) surface, we simulated the STM images of boron sheet on Ag(111) at several different bias voltage. As shown in Fig. 6, the simulated STM images for the buckled triangular borophene display stripes of bright spots in spindle shape at positive voltage. But at the negative bias voltage of − 0.4 V, the simulated STM images show the light and dark stripes, which matches well with experiment result [22]. On the other hand, the simulated STM images of β_12_ borophene maintain the oval shape at both the positive and negative bias voltage. Hence, buckled triangular structure is more likely to be the correct configuration of stripe phase. As for the STM image of χ_3_ borophene, Fig. 6 indicates that the bright spots in all the images come from the boron atoms at the edge of hexagonal vacancies, but their bright contrast changes as the voltage changes from positive to negative. As the bias voltage of 0.2 and − 0.4 V, the brightness of the spots are similar. Additionally, our simulated STM images for the χ_3_’ configuration look similar at bias voltage from 0.8 to − 1.0 V (Fig. 6). They all show the bright spots coming from the boron atoms on the edge of hexagonal vacancies, but only the higher boron atoms are visible and the lower boron atoms in the middle of the unit cell are invisible.

## Conclusions

In summary, we performed first-principles calculations on the atomic structure, stability, and electronic property for the three 2D boron sheets which were grown on the metal surface very recently, namely, buckled triangular, β_12_, and χ_3_ lattice. Our calculations indicate that all the three boron sheets are thermodynamically unstable without the support of metal substrate. The band structures indicate that the buckled triangular boron sheet behaves as a metal with strong anisotropy and β_12_ and χ_3_ boron sheets are also metallic without energy gaps. Additionally, our results show that the energies for the three types of lattices are very close and the lattice match between the buckled triangular and β_12_ boron sheets and Ag(111) surface is quite small. Furthermore, we have found that both buckled triangular and β_12_ boron sheets on the Ag(111) form the rectangular lattice and the parallel striped patterns of STM image but with little difference. Our results provide details to distinguish the two lattices. Most importantly, our simulated STM images give a new explanation to the experimentally observed boron sheets on the Ag(111) surface.

## Abbreviations

2D: Two-dimensional
3D: Three-dimensional
STM: Scanning tunneling microscopy
